# Activin receptor type IIA/IIB blockade increases muscle mass and strength, but compromises glycemic control in mice

**DOI:** 10.1016/j.molmet.2025.102261

**Published:** 2025-09-27

**Authors:** Michala Carlsson, Emma Frank, Joan M. Màrmol, Mona Sadek Ali, Steffen H. Raun, Edmund Battey, Nicoline Resen Andersen, Andrea Irazoki, Camilla Lund, Carlos Henríquez-Olguin, Martina Kubec Højfeldt, Pauline Blomquist, Frederik Duch Bromer, Gabriele Mocciaro, Andreas Lodberg, Christian Brix Folsted Andersen, Marco Eijken, Andreas Mæchel Fritzen, Jonas Roland Knudsen, Erik A. Richter, Lykke Sylow

**Affiliations:** 1 Department of Biomedical Sciences, Faculty of Health and Medical Sciences, University of Copenhagen, Copenhagen, 2200, Denmark; 2 Department of Nutrition, Exercise, and Sports, Faculty of Science, University of Copenhagen, Denmark; 3 Skeletal Muscle Biology, Research and Early Development, Novo Nordisk A/S, Måløv, Denmark; 4 Center for Exercise Physiology and Metabolism, Department of Kinesiology, Faculty of Medicine, Universidad Finis Terrae, Santiago, Chile; 5 Department of Biomedicine, Aarhus University, Aarhus, Denmark; 6 Department of Clinical Medicine, Aarhus University, Aarhus, Denmark; 7 Department of Endocrinology and Internal Medicine, Aarhus University Hospital, Aarhus, Denmark

**Keywords:** Insulin resistance, Activin receptor, Bimagrumab, Obesity, Muscle mass, Glycemic regulation

## Abstract

**Purpose:**

Blocking the Activin receptor type IIA and IIB (ActRIIA/IIB) has clinical potential to increase muscle mass and improve glycemic control in obesity, cancer, and aging. However, the impact of blocking ActRIIA/IIB on strength, metabolic regulation, and insulin action remains unclear.

**Methods:**

Here, we investigated the effect of short- (10 mg kg^−1^ bw, once, 40h) or long-term (10 mg kg^−1^ bw, twice weekly, 21 days) antibody treatment targeting ActRIIA/IIB (αActRIIA/IIB) in lean and diet-induced obese mice and engineered human muscle tissue.

**Results:**

Short-term α ActRIIA/IIB administration in lean mice increased insulin-stimulated glucose uptake in skeletal muscle by 76–105%. Despite this, αActRIIA/IIB-treated mice exhibited 33% elevated blood glucose and glucose intolerance. Long-term αActRIIA/IIB treatment increased muscle mass (+20%) and reduced fat mass (−8%) in obese mice but failed to enhance insulin-stimulated glucose uptake in muscle or adipose tissue. Instead, it induced glucose intolerance, cardiac hypertrophy with glycogen accumulation, and elevated hepatic triacylglycerol and glucose output in response to pyruvate. Concomitantly, long-term αActRIIA/IIB treatment increased strength (+30%) in mouse soleus muscle and prevented activin A-induced loss of tissue strength in engineered human muscle tissue. Surprisingly, long-term α ActRIIA/IIB treatment lowered volitional running (−250%).

**Conclusions:**

Our findings demonstrate that, in accordance with human studies, ActRIIA/IIB blockade holds promise for increasing muscle mass, strength, and muscle insulin sensitivity. However, contrary to the improved glycemic control in humans, ActRIIA/IIB blockade in mice causes severe glucose intolerance and lowers voluntary physical activity. Our study underscores the complex metabolic and functional consequences of ActRIIA/IIB blockade, and highlight species differences on glycemic control, which warrant further investigation.

## Highlights

• ActRIIA/IIB blockade enhances insulin-stimulated glucose uptake.
• αActRIIA/IIB treatment increases force in mouse muscle and engineered human muscle.
• αActRIIA/IIB-treated mice demonstrate reduced voluntary running activity.
• αActRIIA/IIB treatment impairs glycemic control and elevates liver triacylglycerol.
• Species differences in ActRIIA/IIB targeting are critical for preclinical research.

## 1 Introduction

Maintaining muscle mass and glycemic control is vital for human health. However, muscle loss occurs with pharmacologically induced weight loss and is a hallmark of chronic conditions such as cancer, aging, obesity, and type 2 diabetes, contributing to reduced survival [1]. Muscle accounts for up to 75% of insulin-stimulated glucose disposal in humans and is therefore crucial for metabolic regulation [[2], [3], [4], [5], [6]]. Thus, preserving muscle mass and glycemic balance is vital. Despite extensive preclinical and clinical efforts, no approved therapies address both.

Emerging targets to improve both muscle function and glucose homeostasis are the transforming growth factor-β (TGF-β) family ligands, such as activin A and myostatin [7,8]. These ligands inhibit skeletal muscle growth via the activin receptor type IIA and type IIB (ActRIIA/IIB) [9,10]. Conversely, the endogenous activin A and myostatin inhibitor, follistatin, increases muscle mass [[11], [12], [13], [14]], and improves muscle insulin sensitivity towards glucose uptake and protein synthesis in mice [11]. Accordingly, blocking activin A and myostatin promotes hypertrophy and prevents muscle loss, as seen in mice with cancer-induced weight loss [15]. ActRIIA/IIB blockade also mitigate semaglutide-induced muscle loss in obese mice [16]. Yet, understanding the potential ActRIIA/IIB-regulated link between muscle mass regulation and insulin-mediated glucose uptake remains incomprehensible. Moreover, exploring therapeutic interventions addressing muscle metabolism is promising for managing conditions associated with concomitant muscle wasting and insulin resistance [17,18].

Bimagrumab is a human monoclonal antibody that blocks the ActRIIA/IIB [[19], [20], [21], [22]], and has been evaluated in Phase II trials [23,24]. An increased glucose infusion rate during a hyperinsulinemic-euglycemic clamp and reduced HbA1c in insulin-resistant individuals [20,24] suggest that Bimagrumab enhances insulin sensitivity, which is corroborated by a recent study in primates showing reduced HbA1c levels upon myostatin and activin A blockade on top of glucagon-like peptide-1 receptor agonists (GLP1-RA) treatment [25]. However, in mice, emerging evidence suggests that ActRIIA/IIB blockade may disrupt glucose homeostasis, given that hepatic follistatin production caused glucose intolerance [26] and Bimagrumab treatment elevated baseline blood glucose levels [16].

Considering the growing aging population prone to conditions associated with muscle wasting and insulin resistance, such as sarcopenia, cancer, obesity, and diabetes, investigating the effect of ActRIIA/IIB blockade on muscle mass and strength, insulin-mediated glucose uptake, and glycemic control is crucial.

Here, we present data showing that while ActRIIA/IIB blockade holds promise for enhancing muscle mass, function, and insulin sensitivity, it also induces severe glucose intolerance, hepatic steatosis, and reduces voluntary physical activity in mice, underscoring the complex metabolic and functional consequences of ActRIIA/IIB blockade and highlighting the importance of delineating species differences with these potential therapeutics.

## 2 Methods

### 2.1 Animals

All experiments were approved by the Danish Animal Experimental Inspectorate (License: 2021-15-0201-01085). Male C57BL/6JRj mice (Janvier lab, France), were maintained on a 12 h:12 h light–dark cycle and single-housed at thermoneutral temperature (29°C), with nesting and hiding material. A 10 weeks diet intervention was initiated at 14 weeks of age, where lean mice received a standard rodent chow diet 3.1 kcal/g (Altromin no. 1324; Brogaarden, Hørsholm, Denmark) and tap water, and diet-induced obese (DIO) mice received a 45% high-fat 4.75 kcal/g (Research diet no. D12451 2.5 HS; Brogaarden, Hørsholm, Denmark) and 10% sucrose water *ad libitum*.

### 2.2 Body composition analysis

Changes in lean mass and fat mass were determined by quantitative magnetic resonance imaging (MRI) using an Echo MRI scanner (EchoMRI-4in1TM, Echo Medical System LLC, Texas, USA).

### 2.3 αActRIIA/IIB antibody treatment

αActRIIA/IIB antibody (αActRIIA/IIB) was purified from conditioned media using chromatography and exchanged into phosphate – buffered saline (phosphate buffered saline, PBS). The amino acid sequence of the anti-ActRIIA/IIB was identical to the commercial variant known as Bimagrumab. More details about the production and purification of αActRIIA/IIB can be found in [27]. Mice were intraperitoneally (i.p) treated short-term (10 mg kg^−1^ bw,) once for 40h or long-term (10 mg kg^−1^ bw), two times pr week for 21 days with the αActRIIA/IIB.

### 2.4 Glucose tolerance test

Before the test, all mice were fasted for 4h. D-mono-glucose (2g kg^−1^ bw) was administered i.p., and blood was collected from the tail vein. Blood glucose was determined at 0, 20, 40-, 60-, 90-, and 120-minutes post glucose injection using a glucometer (Bayer Contour, Bayer, Switzerland). Plasma from 0 to 20 min was collected, frozen in liquid nitrogen. Insulin levels were measured in duplicates. (Mouse Ultrasensitive Insulin ELISA, #80- INSMSU-E01ALPCO Diagnostics, USA).

### 2.5 Pyruvate tolerance test

The mice were fasted for 4h before sodium pyruvate (1 g kg^−1^ bw) was administered i.p and blood collected from the tail vein. Blood glucose was determined at time points 0, 20, 40, 75, and 90 min using a glucometer (Bayer Contour, Bayer, Switzerland).

### 2.6 *Ex vivo* force assessment

Mice were euthanized by cervical dislocation and soleus muscles were rapidly isolated and non-absorbable 4–0 silk suture loops (Look SP116, Surgical Specialities Corporation) were attached at both ends. The muscles were placed in a DMT Myograph system (820MS; Danish Myo Technology, Hinnerup, Denmark) and incubated at 30°C in a Krebs–Ringer buffer and electrical stimulation protocol was performed as previously described [28].

### 2.7 Insulin tolerance test and *in vivo* 2-deoxy glucose measurements

To determine whole-body insulin tolerance and 2-deoxy-glucose (2DG) uptake in muscle, [3H]2DG (Perkin Elmer) was injected retro-orbitally in a bolus of saline containing 66.7 μCi mL^−1^ [3H]2DG (6 μLg^−1^ bw) in lean and DIO mice, as previously described [29].

### 2.8 Protein extraction and immunoblotting

All tissues were processed, and lysate protein concentration was determined using the bicinchoninic acid method, and immunoblotting was performed as previously described [30].

### 2.9 Liver triacylglycerol content

Liver triacylglycerol content was measured as previously described [31], using commercially available assay kit (Abcam, #ab65336) following manufacturer's instructions.

### 2.10 Glycogen content

Liver and heart glycogen concentrations were determined as glycosyl units after acid hydrolysis by a fluorometric method, as previously described [32].

### 2.11 RNA extraction and real-time-qPCR

RNA from mouse livers was extracted using the RNeasy Mini Kit (Qiagen, #74106) following the manufacturer's instructions as previously described [28]. Primer Sequence: *Pcx_*fwd: CTGAAGTTC CAAACAGTTCGAGG; *Pcx*_ rev: CGCACGAAACACTCGGATG; *Pck1*_ fwd: CTGCATAACGGTCTGGACTTC; *Pck1*_ rev: CAGCAACTGCCCGTACTCC; Housekeeping gene 36b4; fwd: TCA TCC AGC AGG TGT TTG ACA; rev: GGC ACC GAG GCA ACA GTT.

### 2.12 Cross-sectional area analysis

Cross-sectional area (CSA) was assessed as described in [33]. Cryosections were thawed, air-dried, rehydrated, and blocked with 5% goat serum before overnight incubation at 4°C with rabbit anti-Laminin (Merck, L9393; 1:150) followed by a 1-hour incubation at room temperature (RT) with Alexa Fluor 488-conjugated goat anti-rabbit (Invitrogen, A-11008; 1:800). After washing, sections were mounted with Prolong Gold Antifade and imaged using a Zeiss Axioscan.Z1. CSA was quantified by thresholding the Laminin signal in FIJI [34].

### 2.13 Insulin-stimulated glucose uptake human myotubes

Primary myoblasts from Cook Myosite healthy donor 30M were seeded in a 96-well plate (16,000 cells/well) in Myotonic growth media containing MS-3333 MyoTonic™ Growth Supplement and 1% P/S. Following two days of growth, the media was changed to MyoTonic™ differentiation media (MD-5555). On day 4 of differentiation, cells were treated with 1 μM αActRIIA/IIB (Cat #: HY-P99355, MedChem) for 96h. Insulin-stimulated glucose uptake was assessed on day 7 using the Glucose Uptake-Glo Assay (Promega, Cat. #J1343) following the manufacturer's recommendations. In brief, on the day of the experiment, the human myotubes were starved 3h before insulin stimulation. The cells were stimulated with insulin (Novo Nordisk) for 60 min at 37°C, 5% CO_2_. Insulin solution was removed and 50 μL 2DG (10 mM) was added for 10 min and placed on a shaker at RT. Following 2DG transport, 25 μL stop buffer was added and placed on a shaker for 10 min at RT. Then, 25 μL neutralization buffer was added, and the plate was shaken briefly. The 2DG6P detection reagent was added to the plate and incubated for 60 min at RT. After incubation, luminescence was recorded using a ClarioStar Plus (BMG Labtech, Germany).

### 2.14 Generation of engineered muscle tissue from primary human myoblast

Engineered muscle tissues were generated as previously described [35]. In brief, primary myoblasts from Cook Myosite healthy donor 30M (300,000 cells) were resuspended in 42.8 μl Hams F10 medium (Gibco), supplemented with 12 μl Matrigel, 4 μl 50 mg/mL fibrinogen (Sigma–Aldrich), and 1.2 μl 100U/μl thrombin (Sigma–Aldrich) for a total seeding volume of 60 μl, 5 million cells per mL. The cell suspension was applied to a Mantarray casting well with mini-sized wells and a two-post array, and incubated at 37°C, 5% CO2 for 80 min for hydrogel formation. Next, 1 mL of F10 media was added to the wells and, after an additional 10 min incubation, the posts were transferred to Myotonic media (Cook Myosite) supplemented with 5 g/mL aminocaproic acid (Sigma–Aldrich). 24h after casting the engineered muscle tissues, they were transferred to Curi Bio Primary Skeletal Muscle Differentiation media and media was refreshed ever 2–3 days. After 7 days, the engineered muscle tissues were transferred to Curi-Bio Primary Skeletal Muscle Maintenance Media. At day 7, the electrical stimulations began.

### 2.15 Force assessment in engineered muscle tissue

Force production in the engineered muscle tissues was assessed every 2–3 days followed by media change. Assessment was performed using a purpose-built plate lid supporting graphite electrodes compatible with the Mantarray hardware. Force was monitored during electrical stimulation at 1-2-3-5-10-20-30 and 40Hz each at a 2s duration with 8s in between stimulations. Each stimulation consisted of biphasic 75mAmp pulses of 10ms duration [35].

### 2.16 Statistical analysis

Results are shown as mean ± standard error of the mean (SEM) with the individual values shown for bar graphs or mean ± SE for curve graphs. Statistical testing for normally distributed data was performed using t-test, one-way or two-way (repeated measures when appropriate) ANOVA as applicable. Sidak post hoc test was performed for all ANOVAs to test the difference between control and of αActRIIA/IIB treatment. Statistical analyses were performed using GraphPad Prism, version 9 (GraphPad Software, La Jolla, CA, USA, RRID: 002798). The significance level for all tests was set at α < 0.05.

## 3 Results

### 3.1 Short-term ActRIIA/IIB receptor blockade improved muscle insulin sensitivity but caused whole-body glucose intolerance

**Figure 1:**
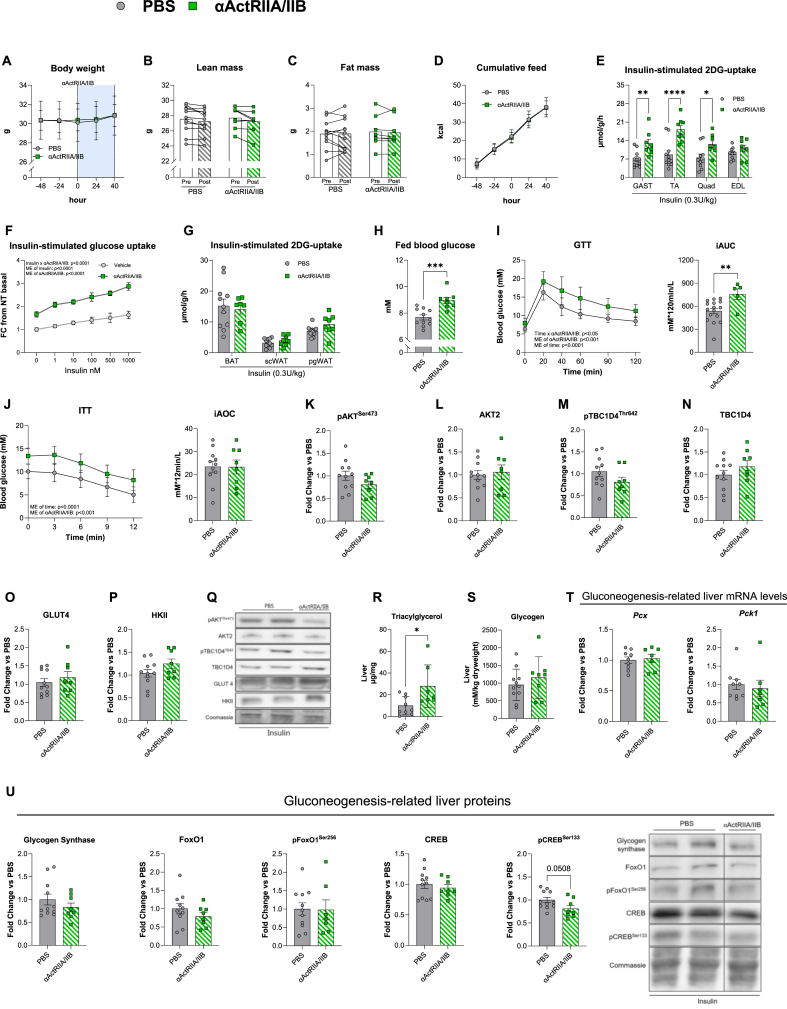
**Short-term ActRIIA/IIB receptor inhibition blockadeby**α**ActRIIA/IIB antibody improves muscle insulin sensitivity, but causes whole-body glucose intolerance. A)** Body weight development in control PBS treated and αActRIIA/IIB treated mice recorded 48h prior to αActRIIA/IIB treatment until 40h after treatment **B)** Magnetic Resonance Imaging-derived lean mass, and **C)** fat mass before (Pre) and 40h after (post) αActRIIA/IIB treatment. **D)** Cumulative kcal intake. **E)** 2-deoxy glucose (2DG) uptake of Gastrocnemius muscle (Gast), Tibialis Anterior (TA), Quadriceps (Quad), Extensor Digitorum Longus (EDL). **F)** Insulin-stimulated glucose uptake in primary human myotubes treated with αActRIIA/IIB for 96h (from day 4 myotubes) to block ActRIIA/IIB signaling. Values are shown as fold change (FC) from non-treated (NT) basal. **G)** 2DG of adipose tissue, Brown adipose tissue (BAT), Subcutaneous white adipose tissue (ScWAT), and Perigonadal WAT (PgWAT). **H)** Blood glucose in the fed state 24 h after αActRIIA/IIBab treatment. **I)** Glucose tolerance test (GTT) (Lean PBS: n = 16 and Lean αActRIIA/IIB: n = 5) with incremental area under the curve (iAUC). Mice that did not respond to the glucose injection (Blood glucose ≤ 10 mM at TP 20 min) were excluded from the dataset. **J)** Insulin tolerance test (ITT) with incremental area over the curve (iAOC). Western blot from gastrocnemius muscle with Protein expression of **K)** phosphorylated (p) pAKT^Ser473^cat#9271, **L)** AKTII cat#3063, **M)** pTBC1D4^Thr642^ cat#4288, **N)** TBC1D4 cat#ab189890, **O)** GLUT4 cat#PA1-1065, **P)** HKII cat#2867, **Q)** Representative blots from western blotting. **R)** Liver triacylglycerol content. **S)** Liver glycogen content. **T)***Pcx* and *Pck1* gene expression levels in liver tissue measured by real-time qPCR. **U)** Western blot from liver tissue of glycogen synthase cat#3886, FoxO1 cat#sc-11350, pFoxO1^Ser256^ cat#9461, CREB cat#4820S, and pCREB^Ser133^ cat#9198S, and representative blot. Lean PBS: n = 8–11 Lean αActRIIA/IIB: n = 8, Values are shown as mean ± SEM including individual values, and as mean ± SD when individual values are not shown. Effect of αActRIIA/IIB: ∗ = p < 0.05, ∗∗ = p < 0.01, ∗∗∗ = p < 0.001.

The short-term effects of αActRIIA/IIB on insulin sensitivity in adipose and skeletal muscle tissues are unknown. To uncover this, we treated lean mice with a single dose (10 mg kg^−1^ bw) of αActRIIA/IIB, which did not influence bw (Figure 1A), lean (Figure 1B) or fat (Figure 1C) mass, or food intake (Figure 1D) 40h post-injection. Yet, insulin-stimulated muscle glucose uptake was nearly doubled in all muscles, except extensor digitorum longus (EDL) (Figure 1E). We next assessed the direct effects of ActRIIA/IIB blockade on insulin-stimulated glucose uptake in primary human myotubes. ActRIIA/IIB blockade elevated glucose uptake 65% at baseline and 75% during insulin stimulation (Figure 1F). These results show that, both in mouse skeletal muscle and human myotubes *in vitro*, short-term ActRIIA/IIB blockade enhanced insulin-stimulated glucose uptake. αActRIIA/IIB's insulin-sensitizing effect seemed selective for the muscle since adipose tissue insulin-stimulated glucose uptake was similar between ActRIIA/IIB groups (Figure 1G). Despite the large enhancement in skeletal muscle glucose uptake, αActRIIA/IIB-treated lean mice exhibited 14% elevated fed blood glucose (Figure 1H) and impaired glucose tolerance (Figure 1I), compared to PBS-treated control mice (n = 5 in αActRIIA/IIB-treated lean mice due to three non-responders during GTT). The insulin tolerance test (ITT) showed a 40% upward shift in blood glucose in αActRIIA/IIB-treated mice, though the incremental area under the curve (iAOC) remained unchanged due to baseline elevated glucose levels (Figure 1J).

To elucidate the mechanisms of enhanced insulin-stimulated muscle glucose uptake, we examined key components of the insulin-signaling pathway [36]. We observed no short-term αActRIIA/IIB-induced alterations in protein content of AKT-TBC1D4 signaling (Figure 1K‒N) or glucose handling proteins GLUT4 (Figure 1O) and hexokinase (HK) II (Figure 1P) in gastrocnemius skeletal muscle, as indicated by representative blots (Figure 1Q). In addition to muscle and adipose tissue, the liver is a major regulator of glycemic control.

Considering the many factors contributing to hepatic glucose output regulation, we measured liver triacylglycerol and glycogen content as well as gluconeogenesis-related genes and proteins. Interestingly, liver triacylglycerol was increased by 2.8-fold in short-term αActRIIA/IIB-treated mice compared to control PBS-treated mice (Figure 1R), while liver glycogen content (Figure 1S) and mRNA expression levels of *Pcx* and *Pck1* (Figure 1T) were unchanged. Liver protein content of Glycogen Synthase, Forkhead box protein O1 (FoxO1), pFoxO1^Ser256^, and cAMP-response-element-binding protein (CREB), were unaffected by short-term αActRIIA/IIB treatment, while pCREB^Ser133^ tended 20% lower (Figure 1U). Thus, short-term αActRIIA/IIB treatment markedly enhanced muscle insulin-stimulated glucose uptake independent of AKT-TBC1D4 muscle insulin signaling but paradoxically elevated blood glucose and induced glucose intolerance.

### 3.2 Long-term αActRIIA/IIB treatment elevated muscle mass and protected against adiposity expansion

**Figure 2:**
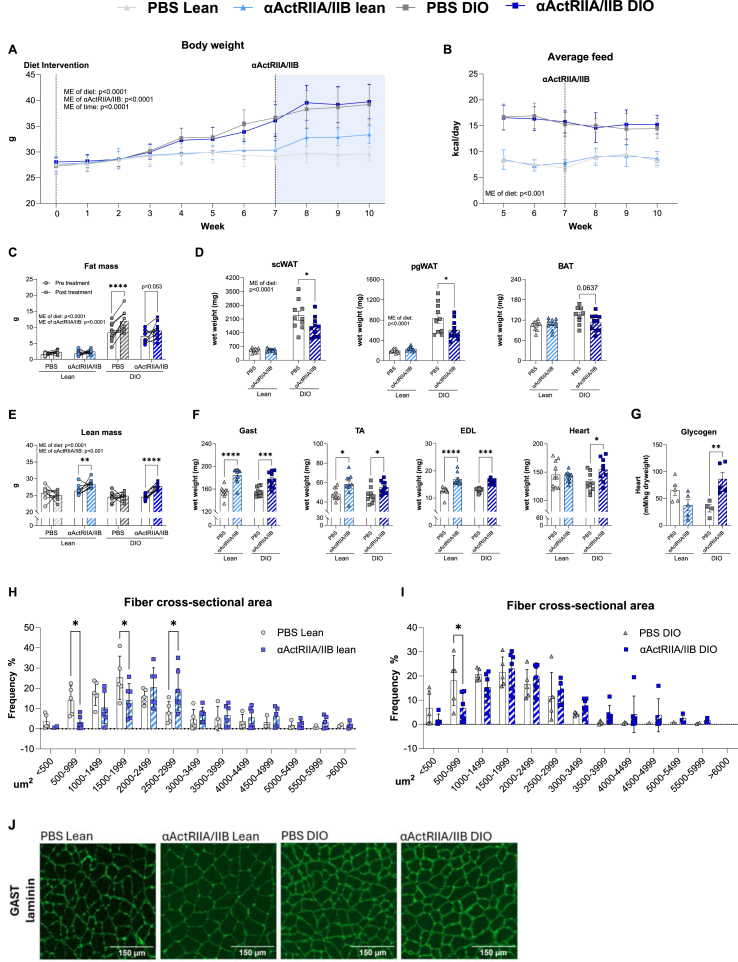
**Long-term**α**ActRIIA/IIB antibody treatment elevates muscle mass and protect against HFHS-induced adiposity expansion. A)** Body weight development before and after diet intervention (control chow diet (Lean) or a high-fat high-sucrose diet (DIO)) and αActRIIA/IIB or PBS treatment. **B)** Average daily caloric intake (excluding sucrose water consumption). **C)** Magnetic Resonance Imaging-derived fat mass. **D)** Weight of subcutaneous white adipose tissue (scWAT), perigonadal WAT (pgWAT), and Brown adipose tissue (BAT). **E)** Lean mass. **F)** whole muscle weights of Gastrocnemius (Gast), Tibialis Anterior (TA), Extensor Digitorum Longus (EDL), and heart. **G)** Heart glycogen content. **H)** Average fiber cross-sectional area distribution in % (single fiber segmentation average 50–300 fibers pr. picture) in Gast of lean mice and **I)** DIO mice. **J)** Representative imaging of laminin-stained Gast muscle fiber slides. PBS Lean: n = 10 αActRIIA/IIB Lean: n = 10 DIO PBS: n = 10, DIO α ActRIIA/IIB: n = 10. For heart glycogen; PBS Lean: n = 5 αActRIIA/IIB Lean: n = 5 DIO PBS: n = 5, DIO α ActRIIA/IIB: n = 5. Values are shown as mean + -SEM including individual values, and as mean + −SD when individual values are not shown. Effect of αActRIIA/IIB: ∗ = p < 0.05, ∗∗ = p < 0.01, ∗∗∗ = p < 0.001

Long-term blockade of the ActRIIA/IIB receptor has been proposed to enhance glucose homeostasis and weight loss while preventing muscle mass loss [[37], [38], [39]], yet results are conflicting [16,26]. To determine the long-term effects of αActRIIA/IIB on whole-body and muscle metabolism, we treated lean and DIO mice with αActRIIA/IIB for 21 days. Expectedly, long-term αActRIIA/IIB treatment increased bw, although only in lean mice (Figure 2A), without altered food intake (Figure 2B). DIO PBS-treated mice displayed a 34 % increase in fat mass while αActRIIA/IIB treatment blunted fat mass gain (Figure 2C), illustrated by reduced overall fat mass and lower weights of subcutaneous white adipose tissue (−18%), perigonadal (−15%), and subscapular brown adipose tissue (−13%) compared to PBS-treated DIO mice (Figure 2D). Lean and DIO mice treated with αActRIIA/IIB displayed increased lean mass by 8% and 13%, respectively (Figure 2E). In both lean and DIO mice, αActRIIA/IIB increased muscle mass by an average 20% (Figure 2F), along with cardiac hypertrophy in DIO mice (18%). The increase in heart weight corroborates an increase in cardiac glycogen content by 2.3-fold in αActRIIA/IIB-treated DIO mice (Figure 2G). Quantification of laminin-stained gastrocnemius muscle sections revealed that αActRIIA/IIB-treated lean mice displayed a 137% increase in the proportion of large fibers within the 2500–2999 μm^2^ range of cross-sectional area compared to PBS-treated controls (Figure 2H). Yet, this effect seemed blunted in αActRIIA/IIB-treated DIO mice (Figure 2I). Representative images are shown inFigure 2J. These results show that long-term αActRIIA/IIB treatment increased muscle mass and prevented adiposity expansion in response to DIO.

### 3.3 ActRIIA/IIB blockade improves tissue strength in mouse soleus and engineered human muscle tissues, but lowers running activity

**Figure 3:**
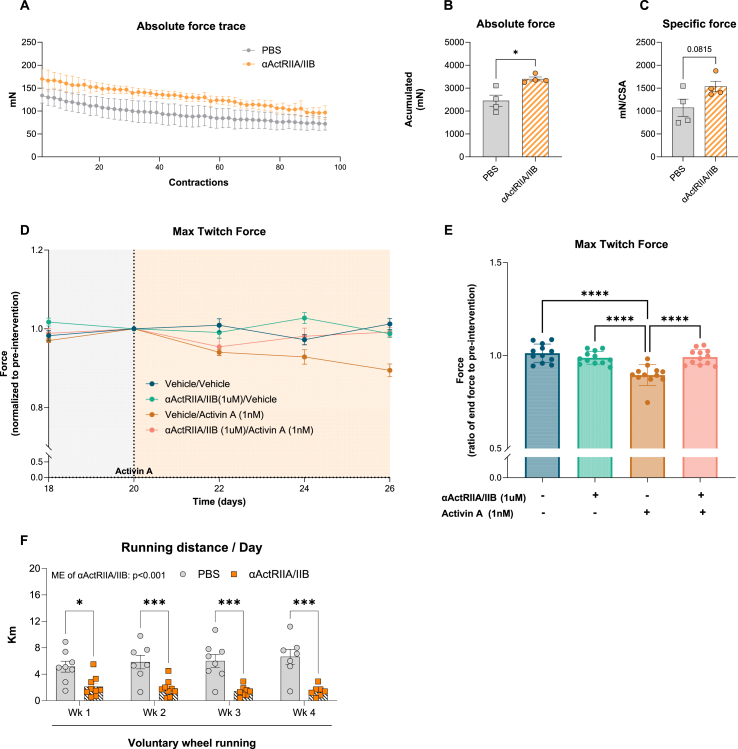
**Mice treated with**α**ActRIIA/IIB antibody display increased muscle force *ex vivo*, but have markedly reduced activity in voluntary wheel running. A)** Absolute force trace of isolated soleus muscles in αActRIIA/IIB or PBS treated mice. Soleus muscles were placed under resting tension (∼ 5 mN) followed by electrical stimulation at 14V and 149.2 Hz with a pulse width and interval of 0.2 ms and 6.5 ms, respectively. Trains of pulses were delivered 75 times with a pause of 5000 ms between pulses, which was repeated 110 times with 2000 ms of pause between pulse trains, allowing for maximal force development. **B)** Accumulated force from absolute force trace **C)** The muscles were measured in length and weight following the electrical stimulation protocol to calculate specific force (wt(mg)/[(length(mm)∗Lf∗1,06]). 14-week-old male mice received two αActRIIA/IIB injections (days 0 and 14), and soleus muscle force was assessed on day 21. **D)** Force assessment in engineered human muscle tissue. Cells were treated with either vehicle or 1 μM αActRIIA/IIB , Cat #: HY-P99355, MedChem). On day 20, activin A (1 nM) was added to the respective groups. Force assessment was performed every 2 days, and force output was normalized to day 20, when activin A treatment began (left panel). Force output normalized to day 20 at the end of the intervention is shown in the right panel. Force was monitored during electrical stimulation at 1-2-3-5-10-20-30 and 40Hz each at a 2sec duration with 8 s in between stimulations. Each stimulation consisted of biphasic 75mAmp pulses of 10 ms duration Media, including treatment groups, was changed every second day. **E)** Voluntary wheel-running intervention. 14-week-old αActRIIA/IIB-treated, and PBS-treated mice were given free access to running wheels for four weeks. Values are shown as + -SEM including individual values, and as mean + −SD when individual values are not shown. Effect αActRIIA/IIB: ∗ = p < 0.05, ∗∗∗ = p < 0.001, ∗∗∗∗ = p < 0.0001.

Having established the beneficial effects of αActRIIA/IIB on muscle mass, we next investigated the effect of αActRIIA/IIB on the functional properties of the muscle, which is a poorly understood but an important clinical outcome. Following long-term αActRIIA/IIB treatment in lean mice, electrically induced force production was increased in the soleus muscles (Figure 3A), for which absolute (Figure 3B) and specific (Figure 3C) force production were both increased 30% (specific force, p = 0.08) by αActRIIA/IIB. To explore the translational clinical relevance of ActRIIA/IIB blockade on muscle function, we examined its effects on force production in engineered muscle tissues. We treated the engineered muscle tissues with activin A (1 nM), αActRIIA/IIB (1 μM), or in conjunction for 6 days (Figure 3D, left panel). Activin A is an endogenous ligand for ActRIIA/IIB, and since αActRIIA/IIB blocks the activin A binding epitope on ActRIIA/IIB, this allowed us to evaluate αActRIIA/IIB's potential to counteract activin A-induced suppression of force production. The presence of activin A lowered force development by 11% in engineered muscle tissues, which was blocked by αActRIIA/IIB (Figure 3D–E), indicating that αActRIIA/IIB improves muscle strength in human muscle by inhibiting activin A binding to ActRIIA/IIB. Thus, in both mouse skeletal muscle and engineered muscle tissues, ActRIIA/IIB blockade elicited positive effects on muscle force production, illustrating the potential for mitigating muscle mass and functional loss.

However, when assessing voluntary physical activity, αActRIIA/IIB-treated lean mice exhibited a marked 250% reduction in voluntary wheel running (Figure 3F). Yet, while the mechanisms underlying αActRIIA/IIB's activity-lowering effects remain unclear, its enhancement of muscle force production in both mouse skeletal muscle and engineered muscle tissues underscores its potential to counteract muscle mass and functional decline.

### 3.4 Long-term αActRIIA/IIB treatment caused hyperglycemia and glucose intolerance without affecting insulin-stimulated glucose uptake or signaling

**Figure 4:**
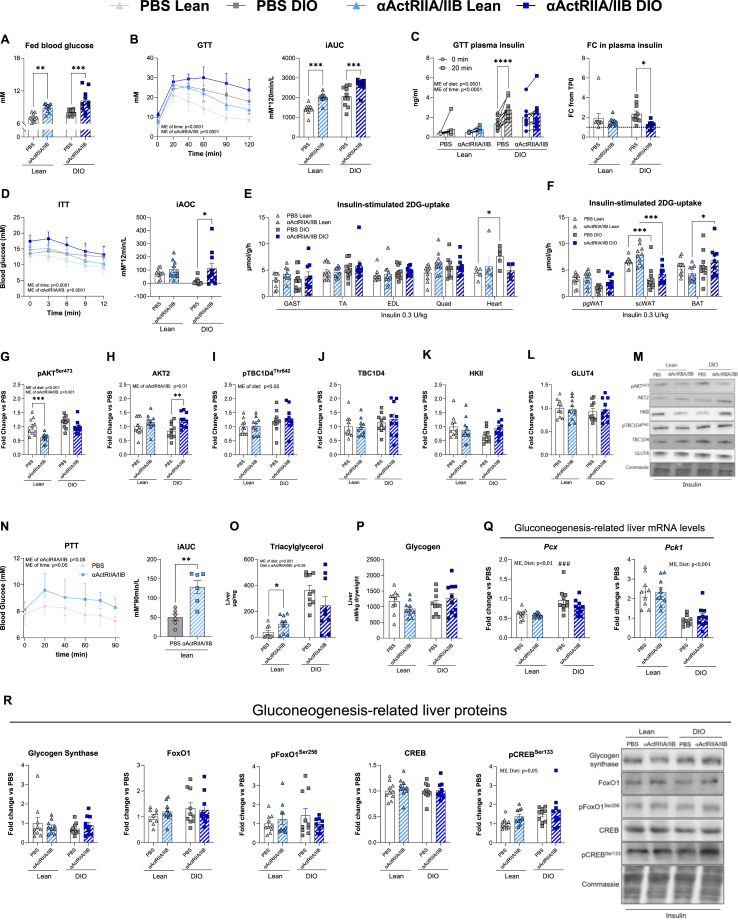
**Long-term**α**ActRIIA/IIB antibody treatment causes whole-body glucose intolerance without effect on glucose uptake and insulin signaling downstreamofA**KT**. A)** Fed blood glucose measured the last week of long-term αActRIIA/IIB treatment. **B)** Blood glucose levels before (0min), 20min, 40 min, 60 min and 90min following an intraperitoneal glucose tolerance test (2 g kg^−1^ body weight), with incremental area under the curve (iAUC) **C)** Plasma insulin from blood drawn at timepoint 0 and after 20 min of the glucose tolerance test, with fold change from TP0. **D)** Blood glucose levels measured at timepoints 0 min, 3 min, 6 min, 9 min, and 12 min following retro-orbital insulin injection (0.3 U/kg body weight) and incremental area over the curve (iAOC) during the 12 min insulin stimulation. iAUC and iAOC were calculated using the trapezoid rule. **E)** 2-deoxy glucose (2DG) uptake of Gastrocnemius muscle (Gast), Tibialis Anterior (TA), Extensor Digitorum Longus (EDL), Quadriceps (Quad), and heart. **F)** 2DG of Perigonadal white adipose tissue (PgWAT), Subcutan WAT (ScWAT), and Brown adipose tissue (BAT). Western blot- Protein expression of **G)** phosphorylated (p) pAKT^Ser473^cat#9271, **H)** AKTII cat#3063, **I)** HKII cat#2867, **J)** GLUT4 cat#PA1-1065, **K)** pTBC1D4^Thr642^ cat#4288, **L)** TBC1D4 cat# ab189890. **M)** Representative blots. **N)** Pyruvate tolerance test performed 7 days after a single αActRIIA/IIB administration. iAUC was calculated from the basal blood glucose concentration determined using the trapezoid rule for the 90 min test. Gene expression levels in liver tissue measured by real-time qPCR. **O)** Liver triacylglycerol content. P) Liver glycogen content. **Q)***Pcx* and *Pck1* gene expression levels in liver tissue measured by real-time qPCR.R) Western blot from liver tissue of glycogen synthase cat#3886, FoxO1 cat#sc-11350, pFoxO1^Ser256^ cat#9461, CREB cat#4820S, and pCREB^Ser133^ cat#9198S, and representative blot. Values are shown as mean + -SEM including individual values, and as mean + −SD when individual values are not shown. Effect of αActRIIA/IIB: ∗ = p < 0.05, ∗∗ = p < 0.01, ∗∗∗ = p < 0.001.

After establishing the beneficial effects of long-term αActRIIA/IIB treatment on body composition, muscle mass, and function, we next examined its impact on glucose homeostasis in lean and DIO mice. We hypothesized that the longer-term beneficial adaptations in body composition would counter the adverse effects of αActRIIA/IIB on glucose tolerance that we observed in response to the short-term treatment. Yet, despite increased muscle mass and lower fat mass, long-term αActRIIA/IIB treatment increased fed blood glucose levels (Figure 4A) and induced marked glucose intolerance in lean mice and exacerbated glucose intolerance in DIO mice (Figure 4B). Plasma insulin levels in lean mice increased approximately 50% in both treatment groups. However, plasma insulin levels increased by 130% in PBS-treated DIO mice, while αActRIIA/IIB-treated DIO mice displayed a modest 20% increase (Figure 4C). Despite lower plasma insulin levels in αActRIIA/IIB-treated DIO mice, we did not detect a defective insulin secretion or islet insulin content and glucose-stimulated insulin release (not shown) as these were comparable between all groups.

As expected, the DIO control mice exhibited insulin resistance, illustrated by a low iAOC for blood glucose in response to insulin, and 4h fasted blood glucose was elevated in αActRIIA/IIB-treated mice (Figure 4D). Yet, the blood glucose-lowering effect of insulin relative to baseline was increased by αActRIIA/IIB treatment, as indicated by iAOC being increased on average by 8-fold in DIO mice treated with αActRIIA/IIB compared to DIO mice treated with PBS. In contrast to the short-term αActRIIA/IIB treatment, the long-term αActRIIA/IIB treatment did not increase insulin-stimulated glucose uptake in skeletal muscle (Figure 4E). Yet, similar to the short-term αActRIIA/IIB treatment, we observed no effect of αActRIIA/IIB on adipose tissue insulin-stimulated glucose uptake (Figure 4F).

Further investigation into molecular insulin signaling revealed that αActRIIA/IIB modestly reduced insulin-stimulated AKT phosphorylation on the serine site 473 (pAKT^ser473^) in lean mice (Figure 4G), while Akt2 protein content was increased (Figure 4H). Despite these alterations in AKT phosphorylation and total AKT2 protein content, the insulin-stimulated phosphorylation of AKT's downstream target, TBC1D4, was unaffected by αActRIIA/IIB treatment (Figure 4I,J). Moreover, we did not observe any difference in HKII (Figure 4K) or GLUT4 (Figure 4L) protein content. Representative blots are shown in (Figure 4M). These data show that long-term αActRIIA/IIB treatment did not affect intramyocellular TBC1D4 signaling or glucose-handling protein content, consistent with unchanged insulin-stimulated glucose uptake. Consequently, altered muscle or adipose insulin sensitivity is unlikely to underlie αActRIIA/IIB-induced hyperglycemia.

Considering the liver's key role in glycemic control, we next assessed the impact of αActRIIA/IIB on hepatic glucose production using a pyruvate tolerance test. Elevated blood glucose levels in lean long-term αActRIIA/IIB-treated mice throughout the test (Figure 4N) suggest enhanced lactate-to-glucose conversion. These results could indicate an increased hepatic gluconeogenesis and glucose output, which could explain the elevated blood glucose and glucose intolerance observed in αActRIIA/IIB-treated mice. Corroborating that, blocking ActRII/B in streptozotocin treated diabetic mice resulted in elevated blood glucose levels compared to control mice when challenged with pyruvate [40]. Similar to short-term αActRIIA/IIB treatment, we observed a 2.6-fold increase in liver triacylglycerol in the chow-fed αActRIIA/IIB-treated mice compared to chow-fed PBS-treated mice. Liver triacylglycerol content was increased by 9.3-fold and 2.3-fold in DIO mice compared to chow-fed PBS-treated and αActRIIA/IIB-treated, respectively. Liver triacylglycerol content was similar between DIO groups (Figure 4O). Despite this, we did not observe alterations in liver glycogen content (Figure 4P), gluconeogenesis-related genes *Pcx* and *Pck1* (Figure 4Q), or in liver content of gluconeogenesis-related proteins that could explain this regulation (Figure 4R).

Together, these results indicate that while αActRIIA/IIB enhances muscle mass and function, αActRIIA/IIB also induces severe glycemic disruptions in mice, likely due to increased hepatic glucose output rather than muscle or adipose insulin resistance.

## 4 Discussion

Here, we investigated the short- and long-term effects of ActRIIA/IIB blockade on insulin sensitivity, skeletal muscle mass and function, and glucose metabolism. Our findings highlight three key outcomes. First, short-term administration of αActRIIA/IIB enhanced insulin-stimulated glucose uptake in mouse and primary human muscle cells, an effect that was not sustained with long-term αActRIIA/IIB treatment. Second, αActRIIA/IIB treatment increased muscle mass and improved absolute force production in isolated murine soleus muscle and engineered muscle tissues. Third, despite these benefits, αActRIIA/IIB-treated mice exhibited markedly elevated fasting blood glucose levels and glucose intolerance likely due to increased hepatic glucose output.

Our first key finding was that short-term αActRIIA/IIB administration enhances insulin-stimulated glucose uptake in mouse skeletal muscle and primary human muscle cells. Our data aligns with evidence showing that inhibition of ActRIIA/IIB responsive ligands such as myostatin or activin A and GDF11 via muscle follistatin overexpression improves insulin sensitivity [11,41]. In contrast to muscle, αActRIIA/IIB did not alter adipose tissue insulin-stimulated glucose uptake in neither lean nor obese mice, suggesting that the insulin-sensitizing effect of short-term αActRIIA/IIB is muscle-selective. However, the elevated insulin-stimulated glucose uptake by αActRIIA/IIB was transient and not sustained with long-term treatment, which warrants further investigation. αActRIIA/IIB treatment may have induced a general increase in glucose uptake. While basal uptake was not assessed in mice, both basal and insulin-stimulated uptake were elevated in primary human myotubes. Such overall increases could explain the lack of enhanced insulin signaling in mouse muscle, suggesting insulin-independent mechanisms. In the heart, we observed glycogen accumulation in DIO αActRIIA/IIB–treated mice. Similarly, in rodents and rhesus monkeys, MK-8722–mediated AMPK activation drove strong insulin-independent glucose uptake in muscle, but also caused cardiac hypertrophy and glycogen build-up [42]. These changes may impair cardiac function [43] and our results that αActRIIA/IIB can lead to cardiac glycogen accumulation underscore the need for careful evaluation in humans.

Our second key finding documents that long-term αActRIIA/IIB-treatment can improve muscle force production. This was evidenced in both isolated mouse skeletal muscle following *in vivo* αActRIIA/IIB treatment, as well as engineered human muscle tissues. These results indicate that blocking the ActRIIA/IIB pathway, well-documented to increase muscle mass [16,37,44], also has beneficial effects on muscle functional capacity, supporting previous findings of improved exercise capacity and 6-min walking test in Bimagrumab-treated mice and humans, respectively [16,45]. Surprisingly, despite these improvements in muscle force generation, we found that αActRIIA/IIB-treated mice demonstrated markedly reduced voluntary wheel-running activity. In line with our observations, participants from clinical trials reported muscle myalgia when taking Bimagrumab [46]. Future studies should aim to elucidate the mechanisms underlying ActRIIA/IIB blockade–associated inactivity in mice and myalgia in humans.

Lastly, we were surprised by our third major finding, that despite improvements on body composition, muscle mass and function, αActRIIA/IIB-treated mice exhibited markedly elevated fed- and fasting blood glucose levels, and glucose intolerance. These findings align with emerging evidence from mice studies suggesting that ActRIIA/IIB blockade may disrupt glucose homeostasis, given that hepatic follistatin production caused glucose intolerance [26] and αActRIIA/IIB treatment caused elevated blood glucose levels in fed mice [16,40], similar to our findings. However, these results are in contrast to clinical data showing reduced HbA1c in Phase II clinical trials with a single dose of Bimagrumab [20,24], or reduced HbA1c levels upon long-term myostatin and activin A inhibition in primates co-treated with GLP-1RA [25]. These discrepancies remain to be resolved but could be due to species-specific effects and/or differences in dosing. Given the extensive use of mice in research, determining whether divergent results arise from species differences is essential for selecting appropriate preclinical models.

In our study, the detrimental effects of αActRIIA/IIB treatment on glucose handling were not due to insulin resistance of the skeletal muscle or adipose tissue. Instead, our findings suggest increased hepatic gluconeogenesis, indicated by enhanced lactate-to-glucose conversion in pyruvate tolerance tests and possibly driven by elevated liver triacylglycerol. This may contribute to higher blood glucose, consistent with the link between hepatic triacylglycerol and gluconeogenesis in humans [47]. In contrast to our findings in mice, patients treated with Bimagrumab exhibited decreased hepatic fat fraction compared to placebo-treated controls. Our data suggest a mouse-specific outcome of αActRIIA/IIB treatment concerning glycemic control and liver fat metabolism.

## 5 Conclusion

Together, these results indicate that, while αActRIIA/IIB enhances muscle mass and function, αActRIIA/IIB also induces cardiac hypertrophy with glycogen accumulation in DIO mice and causes severe glycemic disruptions in mice, likely due to increased hepatic glucose output and triacylglycerol accumulation.

These findings underscore the need to consider the metabolic consequences of ActRIIA/IIB blockade when developing therapies for conditions such as sarcopenic obesity, cancer, and diabetes. Our results also indicate species-specific differences in response to αActRIIA/IIB, highlighting the importance of this consideration in future research.

## CRediT authorship contribution statement

**Michala Carlsson:** Writing – review & editing, Writing – original draft, Visualization, Validation, Project administration, Methodology, Investigation, Formal analysis, Data curation, Conceptualization. **Emma Frank:** Writing – review & editing, Writing – original draft, Visualization, Validation, Methodology, Investigation, Formal analysis, Data curation, Conceptualization. **Joan M. Màrmol:** Writing – review & editing, Writing – original draft, Project administration, Methodology, Data curation. **Mona Sadek Ali:** Investigation. **Steffen H. Raun:** Investigation, Data curation. **Edmund Battey:** Writing – review & editing, Writing – original draft, Software, Methodology, Data curation. **Nicoline Resen Andersen:** Writing – review & editing, Investigation, Data curation. **Andrea Irazoki:** Writing – review & editing, Supervision, Methodology, Investigation, Data curation. **Camilla Lund:** Writing – review & editing, Data curation. **Carlos Henríquez-Olguin:** Writing – review & editing, Investigation, Data curation. **Martina Kubec Højfeldt:** Methodology, Investigation. **Pauline Blomquist:** Writing – review & editing, Investigation, Conceptualization. **Frederik Duch Bromer:** Writing – review & editing, Methodology. **Gabriele Mocciaro:** Data curation, Writing – review & editing. **Andreas Lodber:** Writing – review & editing, Validation, Resources, Project administration, Conceptualization. **Christian Brix Folsted Andersen:** Writing – review & editing, Investigation, Conceptualization. **Marco Eijken:** Writing – review & editing, Investigation, Conceptualization. **Andreas Mæchel Fritzen:** Data curation, Writing – review & editing. **Jonas Roland Knudsen:** Writing – review & editing, Resources, Methodology, Conceptualization. **Erik A. Richter:** Writing – review & editing, Resources, Methodology, Investigation, Conceptualization. **Lykke Sylow:** Writing – review & editing, Writing – original draft, Visualization, Validation, Supervision, Resources, Investigation, Funding acquisition, Formal analysis, Conceptualization.

## Declaration of Generative AI and AI-assisted technologies in the writing process

Statement: During the preparation of this work, the authors used ChatGPT in order to do minor corrections and shortening of sentences. After using this tool/service, the authors reviewed and edited the content as needed and take full responsibility for the content of the published article.

## Funding

The 10.13039/100008392Danish Council for Independent Research, Medical Sciences (grant DFF-4004-00233 to L.S.). The 10.13039/501100009708Novo Nordisk Foundation (grant NNF16OC0023418 and NNF18OC0032082 to L.S.).

## Declaration of competing interest

CH-O, PB, MHH, and JRK are employed at Novo Nordisk S/A. Andreas Lodberg has served as a consultant or has received advisory fees from Acarios, Aureka Biotechnologies, Bluejay Therapeutics, Epirium Bio, and Morgan Stanley. Andreas Lodberg has performed sponsored research for Keros Therapeutics.

## Data Availability

Data will be made available on request.
